# Parallel ecological networks in ecosystems

**DOI:** 10.1098/rstb.2008.0222

**Published:** 2009-06-27

**Authors:** Han Olff, David Alonso, Matty P. Berg, B. Klemens Eriksson, Michel Loreau, Theunis Piersma, Neil Rooney

**Affiliations:** 1 Centre for Ecological and Evolutionary Studies, University of GroningenPO Box 14, 9750 AA Haren, The Netherlands; 2 Department of Animal Ecology, Institute of Ecological Science, Vrije UniversiteitDe Boelelaan 1085, 1081 HV Amsterdam, The Netherlands; 3 Department of Biology, McGill UniversityMontreal, Québec H3A 1B1, Canada; 4 Department of Marine Ecology, Royal Netherlands Institute for Sea Research (NIOZ)PO Box 59, 1790 AB Den Burg, Texel, The Netherlands; 5 Department of Integrative Biology, University of GuelphGuelph, Ontario N1G 2W1, Canada

**Keywords:** food webs, predator–prey interactions, ecological networks, non-trophic interactions, ecosystem engineers, ecological stoichiometry

## Abstract

In ecosystems, species interact with other species directly and through abiotic factors in multiple ways, often forming complex networks of various types of ecological interaction. Out of this suite of interactions, predator–prey interactions have received most attention. The resulting food webs, however, will always operate simultaneously with networks based on other types of ecological interaction, such as through the activities of ecosystem engineers or mutualistic interactions. Little is known about how to classify, organize and quantify these other ecological networks and their mutual interplay. The aim of this paper is to provide new and testable ideas on how to understand and model ecosystems in which many different types of ecological interaction operate simultaneously. We approach this problem by first identifying six main types of interaction that operate within ecosystems, of which food web interactions are one. Then, we propose that food webs are structured among two main axes of organization: a vertical (classic) axis representing trophic position and a new horizontal ‘ecological stoichiometry’ axis representing decreasing palatability of plant parts and detritus for herbivores and detrivores and slower turnover times. The usefulness of these new ideas is then explored with three very different ecosystems as test cases: temperate intertidal mudflats; temperate short grass prairie; and tropical savannah.

## 1. Introduction

Ecology was first defined in 1869 as the ‘study of the interaction of organisms with their environment’ (Haeckel 1869, quoted in Begon *et al*. 1990) and later as ‘the scientific study of the distribution and abundance of organisms’ (Andrewartha 1961). Krebs (2001) combined these definitions into the ‘scientific study of the interactions that determine the distribution and abundance of organisms’. He did not use the word ‘environment’, because it is already inclusive in the definition. The environment of an organism consists of all those phenomena outside an organism that influence it, whether those factors are physical (abiotic) or are other organisms (biotic). Hence the ‘interactions’ in the definition of Krebs are the interplay of organisms with these biotic and abiotic factors (Begon *et al*. 1990).

For over a century now, ecologists have been describing the patterns in the distribution (Lomolino *et al*. 2005) and the abundance (McGill 2006; McGill *et al*. 2007) of organisms. With respect to the study of interactions (the explanatory part of ecology), consumer–resource interactions have received by far most empirical and theoretical study, both from a single trophic (Tilman 1982) and from a multitrophic, food web perspective (Cohen 1978; DeAngelis 1992; Polis & Winemiller 1996). Studies that use food web theory to better understand a particular ecosystem thus implicitly assume that predation is the most important process that regulates the abundance of organisms in that ecosystem (Berlow *et al*. 2004).

However, it has long been recognized that species interact in ecosystems with other species and with abiotic factors in many ways, of which predator–prey interactions are only one possibility (Hutchinson 1959). For example, organisms interact with other species through producing resources such as detritus and mineral nutrients and through non-trophic interactions (e.g. pollination, production of toxicants). Also, organisms can show strong interactions with abiotic (non-resource) conditions. In addition, relevant interactions that affect organisms include various spatial interactions (exchange of organisms, materials and energy), external environmental forcing, as well as various physical and chemical interactions that operate within ecosystems.

These days, ecologists are increasingly challenged to better understand and predict the impacts of human activities on biodiversity and the functioning of ecosystems, such as the consequences of harvesting populations (forestry, fisheries), modification of material cycles (e.g. eutrophication) and human-induced climate change. Key general questions in this conservation agenda are: (i) which (types of) species will be most vulnerable to extinction in the near future, (ii) are ecosystems of high biodiversity (such as tropical forests, coral reefs) under greater threat than those less diverse, (iii) will the loss of some species (e.g. top predators) lead to cascading losses of other species, and impair the functioning of ecosystems, (iv) should some species therefore be given special attention in conservation schemes, (v) how will the human disruption of natural element cycles and the introduction of novel chemical compounds and non-native species affect the functioning of natural ecosystems and impair the services they provide to us, and (vi) what will be the consequences of emerging (zoonotic) diseases? All these questions will affect the abundance and distribution of species, with associated effects on the functioning of ecosystems. Answers to these questions are urgently needed to set conservation priorities and take appropriate action to restrict biodiversity loss due to human-driven environmental change.

Since the pioneering work of Elton (1927), Lindeman (1942) and Hairston *et al*. (1960), the field of food web theory has developed into a central concept in ecology. It is therefore a logical field to turn to first for answers to the above conservation-oriented questions, as it aims to understand the abundance and distribution of organisms from the perspective of species interactions. Indeed, the central questions addressed in food web ecology seem highly relevant for conservation and management. For example, what is the effect of increased nutrient supply on trophic web structure (Carpenter & Kitchell 1993; Scheffer & Carpenter 2003)? Or, how does the diversity and complexity of food webs affect their stability, e.g. the extent to which small perturbations in some species lead to the loss of other species (May 1973; Dunne *et al*. 2002; Ives & Carpenter 2007; Neutel *et al*. 2007)? What determines whether the loss of top predators leads to cascades of secondary extinctions (Scheffer *et al*. 2005; Borrvall & Ebenman 2006; Otto *et al*. 2008)? However, in a recent list of 100 ecological questions of high policy relevance in the UK (Sutherland *et al*. 2006), the word ‘food web’ or ‘interaction web’ did not occur once, suggesting it is not, or at least not perceived this way.

In our view, this ‘struggle for relevance’ of food web ecology is due to two main problems. Firstly, food webs consist of a ‘road map’ of predator–prey interactions in ecosystems. However, species in ecosystems interact with each other and with their environment in many other ways than through consumer–resource interactions. These ‘other interactions’ have been insufficiently acknowledged and studied from a network perspective, ‘pushing’ conservation-oriented research often towards a species-centred approach (in which all such interactions are included for a particular species). However, in such species-centred research, the operation of the key indirect effects among species that characterize ecological networks are probably missed. Inclusion of non-trophic interactions broadens food web studies to the analysis of interaction webs.

Secondly, food web studies have often been too system specific, and we need a more general ‘template’ of functional classification of species along main axes of organization (not only trophic position) in food webs to be able to make comparisons between different ecosystems, and to study the interplay of networks based on consumer–resource interactions with networks based on other types of interaction that operate within the same ecosystem.

The goal of this paper is to contribute to the solutions for both problems. First, we briefly discuss the general principles behind the organizational forces at work in ecological interaction webs. Then, we propose six main types of ecological interaction that operate (often simultaneously) in ecosystems, each of which, or combinations of which, will form separate networks of interactions. These parallel ecological networks functionally link to each other through the species as network nodes. Consumer–resource interactions, leading to food webs, are one of those possible networks, and an important, basic one, but is not the only one. We continue by proposing that food webs are organized along two main dimensions: their ‘classic’ vertical dimension that reflects the trophic position of species, and a newly proposed horizontal ‘stoichiometric’ axis, representing decreasing palatability of plant parts and detritus for herbivores and detrivores (driven by evolutionary radiation between autotrophs in competition for light). The main goal of identifying both the six main interaction types and the above two axes of food web organization is to provide a framework and general notation that can be used to describe interaction webs across very different ecosystems. We qualitatively explore this framework by unravelling the parallel interaction webs that operate in three very different ecosystems: European intertidal mudflats; North American short grass prairie; and African savannah. For each ecosystem, we draw the parallel interaction webs for two or three main types of interaction, such as consumer–resource interactions and interactions between species and abiotic (non-resource) conditions. We finish by discussing future directions in the analysis of the interplay between parallel ecological networks in ecosystems, and some conservation implications of their joint operation.

## 2. Ecological interaction webs as complex adaptive systems

In his excellent treatise on the philosophical foundations of interaction web studies, Ulanowicz (1997) makes important points on the nature of causality and the importance of conditional probabilities. First, he emphasizes that ecological interaction webs belong to the larger class of complex adaptive systems, which means that causes and explanations arise not only from lower levels of organization (e.g. from ecophysiology, behavioural ecology, population ecology), but also at the focal level of organization (see also Levin 1998; Morowitz 2002). This makes system behaviour, especially on longer time scales, to some degree, autonomous with respect to lower level events (Allen & Starr 1982). The study of complex adaptive (or dynamic) systems has a long tradition in physics and chemistry (Holland 1999). However, the main insights from these fields may have relatively little relevance for biological organisms and the way they grow and function, and organize into interaction webs and ecosystems, due to the unique regulatory role of DNA and the operation of evolution by natural selection (Werner 2007).

For those causes arising at the focal level of ecological interaction webs, the challenge is to discover the principles that govern their organization, or, ‘how lots of things are put together in the same place’ (Ulanowicz 1997). This challenge is shared with other fields in the life sciences, for example, with developmental biology, where the main ‘grammar’ of the genetic code still mostly awaits discovery, now that the translation of ‘letters and words’ is available (Lewin 1984; Barbieri 2002). The emerging field of systems biology (Kitano 2002) now aims at unravelling exactly how the network of interactions among genes, proteins, organelles, cells and tissues within organisms forms this grammar.

The general scientific problem here is that causes of organization in ecological networks (and other complex adaptive systems) arise through *conditional probabilities*, which means that all probabilities (e.g. likelihood of change in the abundance of a species) are always contingent to a greater or lesser extent upon local and historic circumstances and interfering events (Ulanowicz & Wolff 1991). For example, the dynamics of three species in a trophic chain can radically change if species 3 evolves a trait that promotes species 1, causing an indirect mutualism. In this case, species 2 will be promoted, even though it did not change its behaviour or physiology at all (Ulanowicz 1995). Also, a predator–prey interaction will have a different effect on either population if the prey has to compete with another prey (leading to apparent competition), or, if the predator is a prey itself to another predator (intraguild predation). And, some species of prey may use phenotypic flexibility to directly adjust their phenotype in the presence of specific predators (Werner & Peacor 2003), while in other cases predators adjust their phenotype in the presence of specific prey (Piersma & Lindstrom 1997; Piersma & Drent 2003). In both cases, such phenotypic adjustments will have consequences for other consumer–resource interactions that the species is involved in. The reasons for the absence or presence of such interfering species may even lay outside the current spatial and temporal domain of observation, due to historical or geographical factors (Ricklefs & Schluter 1993). Dealing with such conditional probabilities requires a redefinition of classic mechanisms (causes imposed by lower level of organization and system components in a deterministic, ‘Newtonian’ way). Evolutionary biologists face similar problems in deducting how organization arises through the operation of conditional probabilities of change, e.g. when developing theory for adaptive dynamics (Dieckmann & Metz 2001) and coevolutionary dynamics (Thompson 2005).

The now widely recognized general feature of complex adaptive systems is that the prevalence of strong conditional probabilities does not necessarily lead to unpredictable, chaotic or erratic structures and dynamics. Instead, emergent structural properties and behaviour often arise at the system level (Levin 1998; Holland 1999; Morowitz 2002), pointing at an underlying ‘semantics’ of system organization (Barbieri 2002). For food webs, such regularities arise for example in their topological organization (Pimm 1982; Williams & Martinez 2000; Montoya *et al*. 2006; Bascompte 2007), the organization of flows, thus interaction weights (Ulanowicz 1997; Neutel *et al*. 2002; Rooney *et al*. 2006; Neutel *et al*. 2007) or their spatial organization (McCann *et al*. 2005). However, clear rules and principles about ‘how lots of things are put together’ in food webs still await description (Ulanowicz 1997).

Insights into specific ‘few-species-interaction-configurations’, or modules (Menge 1995; Holt 1997; Bascompte & Melian 2005) for consumer–resource interactions have much increased over the last decades. For example, we know much more now about resource competition (Schoener 1974; Tilman 1982), mutualism (Oksanen 1988), apparent competition (Holt 1977), indirect mutualism (Vandermeer 1980; Ulanowicz 1997), intraguild predation (Polis *et al*. 1989), positive interactions such as facilitation (Callaway 2007), positive feedbacks (DeAngelis *et al*. 1986), regulatory feedbacks (Bagdassarian *et al*. 2007), trophic cascades (Carpenter *et al*. 2008) and multiple stable state dynamics (Scheffer & Carpenter 2003). These may all be considered organizational forces that structure networks, but all may not be of equal importance. For example, Ulanowicz (1997) makes a strong case for the special importance of indirect mutualism as an organizational force in food webs, as the resulting feedback loops ‘attract’ resources towards them.

But how such modules together organize into complex interaction webs remains as yet largely unresolved, especially for types of interaction other than between consumers and resources. Some progress has been made in the field of food webs, trying to capture organization in concepts such as ascendancy, which quantifies the growth and development in a network due to indirect mutualism (Baird & Ulanowicz 1989; Ulanowicz 1997; Baird *et al*. 2007), as well as in the study of evolutionary networks using graph theory (Lieberman *et al*. 2005). Owing to their predominance of conditional probabilities, the study of ecological networks is more complex than ‘adding up’ the ecophysiology, population biology and behavioural ecology of the component species as promoted for a long time (Schoener 1986). We also need to identify much better the processes that arise at the level of interaction webs. Although some of the emergent properties of complex communities and ecosystems have now been established as macroecological rules and patterns in the distribution and abundance of organisms (Brown & Maurer 1989; McGill *et al*. 2007), we feel we have yet not been able yet to identify most of the underlying organizational principles that govern these rules and patterns. We suggest that this is caused by too little study of non-trophic ecological networks that operate in parallel to consumer–resource networks, and also by the lack of a good organizational framework to compare interaction webs across ecosystems.

## 3. Six main types of interaction in ecosystems

Current food web theory is not well equipped to deal with the changes in environmental factors (such as temperature or pH) towards which the species in the web may be differentially adapted (Raffaelli 2006), or to make predictions for ecosystems where interactions of organisms with their abiotic environment play a major role in addition to trophic interactions. Although further work on networks of just predator–prey interactions (food webs) is needed, we agree with Berlow *et al*. (2004) that we now need a rigorous framework to determine how and which processes should be included in food web theory out of the growing set of possible ecological interactions that is considered to be important. This can be seen as a generalization of food web theory to a theory that covers ecological networks in general. This fits with recent studies trying to combine nutrient flows between ecosystems with trophic interactions in meta-ecosystem theory (Polis *et al*. 1997; Loreau *et al*. 2003), trophic and non-trophic interactions in interaction web theory (Arditi *et al*. 2005; Bascompte 2007; Dambacher & Ramos-Jiliberto 2007; Goudard & Loreau 2008), dispersal limitation and competition in metacommunity theory (Leibold *et al*. 2004), trophic interactions with species–environment feedback (Bagdassarian *et al*. 2007) and dispersal, sampling processes and speciation in neutral biodiversity theory (Hubbell 2001).

**Figure 1:**
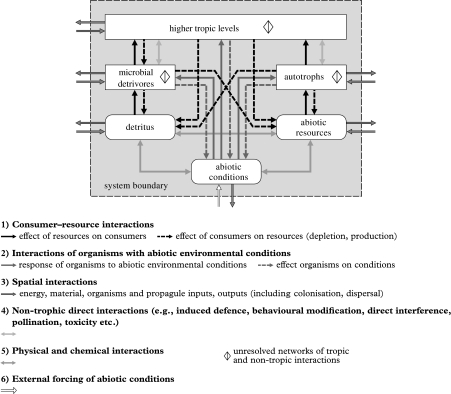
A proposed general framework of ecological networks, indicating the dual detrital versus primary producer pathways of energy and nutrient flow that sustains higher trophic levels. Boxes represent basic compartment types or factors, and different types of arrow represent six main types of interaction that structure ecological networks. Some compartments may contain an unresolved web of interactions, based on consumer–resource interactions and non-trophic direct interactions.

Repeating that ecology is both about jointly understanding interactions among organisms, and between organisms and their abiotic environment, we propose six main types of ecological interaction that operate in ecosystems, with a general framework for their topological connection among six basic ecosystem compartments (figure 1). These six types of interaction are: (i) consumer–resource interactions, (ii) interactions between organisms and abiotic (non-resource) conditions, (iii) spatial interactions (inputs and outputs of energy, nutrients, organisms), (iv) non-trophic direct interactions among organisms, (v) physical and chemical interactions among factors/compartments, and (vi) external forcing of abiotic conditions. These six types of interaction potentially operate among three biotic and three abiotic basic compartments (figure 1). The abiotic compartments are (i) abiotic resources (such as light, nitrate, ammonium, phosphate) that are consumed and depleted by autotrophs, (ii) abiotic conditions, that affect both autotrophs and heterotrophs but are not consumed or depleted by them (such as salinity, soil texture, sediment aeration, soil and water pH, temperature) but that can be modified (e.g. by ecosystem engineers (Jones *et al*. 1994; Lawton 1994)) and (iii) detritus (non-living organic material). The main three biotic compartments are (i) autotrophs that can harvest their own energy, either from light or chemical sources, (ii) microbial detrivores that break down detritus into its mineral components, thus producing resources for autotrophs and (iii) higher trophic levels that consume autotrophs, microbial detrivores and/or each other, and mineralize nutrients for autotrophs. This interaction-web framework builds on earlier ideas for marine systems by Azam *et al*. (1983) and for terrestrial systems by Wardle (2002), Moore *et al*. (2004), Bardgett (2005) and others, who all emphasized the importance of the ‘dual foundation’ of food webs on both autotrophs (plants, photosynthetic or chemoautotrophic microbes) and microbial detrivores, but adding the effect of environmental (non-resource) conditions. The autotroph- versus detritus-based side of figure 1 can be viewed as two alternative channels that provide energy to higher trophic levels, while being strongly functionally connected at the bottom through the process of energy fixation (a ‘service’ of the autotrophs facilitating the development of the detritus-based side) and element recycling (a ‘service’ that especially the microbes on the detritus-based side provided to the autotrophs). Depending on the ecosystem type, these two main energy channels are usually still separate at low trophic levels (e.g. plant- versus microbial detrivore-based grazers), while becoming more connected at higher trophic levels, where omnivorous predators often receive energy through both channels. In very open ecosystems that receive their energy through detritus imports, such as tree holes (Kitching 1971), deep oceans systems (Andersson *et al*. 2004), streams or shaded lakes, the food web can be almost entirely detritus based. In more closed ecosystems on the other hand, autotrophs generally require microbial detrivores to recycle mineral nutrients (called the microbial loop in pelagic systems) and the food web will receive energy through both channels. It should be noted that the three ‘biotic boxes’ in figure 1 aggregate complex trophic interactions through unresolved ecological networks. Each of these boxes can also be expanded to networks of higher detail (e.g. in functional groups such as herbivores consuming plants, predators of herbivores, predators of predators of herbivores, pathogens, pollinators, etc., or down to the species level).

As listed in figure 1, we suggest that up to six main types of direct interaction can operate simultaneously in any ecosystem, where it is not *a priori* clear which ones will dominate in determining community structure and ecosystem functioning. Consumer–resource interactions are of course a basic one (each species generally has to eat), but such food web interactions will be affected by other types of interaction that operate in the ecosystem at the same time. When these other interactions involve only one or two species, this may still be ‘fixed’ by modifying food web models to include such effects (Arditi *et al*. 2005; Goudard & Loreau 2008). However, when the other types of interaction result in ecological networks as well (of which we will show examples later), this requires a different approach, the here-proposed analyses of ‘parallel ecological networks’. Before we continue with this discussion, we first identify and discuss each of the six main interaction types that we suggest are structuring ecosystems.

### (a) Consumer–resource interactions

Resources are all things consumed by an organism (Tilman 1982). Not only are such resources incorporated in the body, they also represent quantities that are reduced by the activities of the organism without actual ingestion (Begon *et al*. 1990). Where nitrate, phosphate and light are resources for a plant, so are nectar, pollen and a hole in a log resources for a bee, and acorns, walnuts, other seeds and a larger hole in a log resources for a squirrel (Tilman 1982). Basic approaches for modelling and measuring classic consumer–resource interactions are extensively reviewed elsewhere (Lotka 1932; May 1973; Schoener 1974; Pimm 1982; Tilman 1982; de Ruiter *et al*. 1995; Berlow *et al*. 2004; Wootton & Emmerson 2005); we will not repeat them here. As outlined by Holt (1997), many indirect trophic interactions such as resource competition, mutualism and trophic cascades can be viewed as manifestations of a particular topological arrangement of multiple consumer–resource interactions, which he termed community modules. For example, pollination can be viewed as a bidirectional consumer–resource interaction but with a reward in a different currency for each partner (energy versus information), similar to a plant–mycorrhizae association (nutrient, water versus energy).

**Figure 2:**
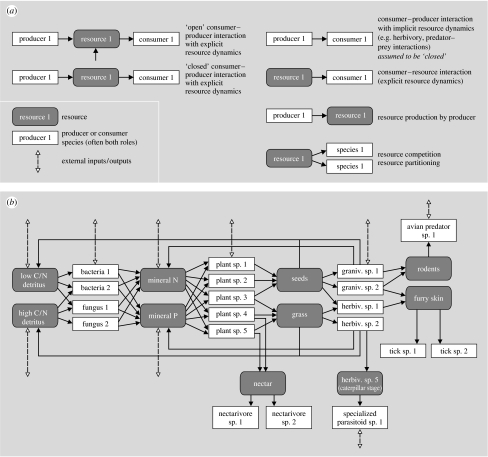
(*a*) Definition of basic interaction types between producers, resources and consumers and (*b*) an example ecological network based on these interactions. The key point is the separation between species, and the resources that they produce and consume. These resources can be assessed implicitly and explicitly. Multiple species can contribute to the same resource compartment or class, and multiple consumers can exploit resources, irrespective of the species that contributed to or forms the resource compartment. graniv., granivorous; herbiv, herbivorous; sp, species.

Consumer–resource interactions form the backbone of food webs in which consumers interact with their resources through ingestion (predator–prey interactions). However, the definition of resources above implies one very important (often missed) point: most food webs cover only a subset of all consumer–resource interactions that operate in an ecosystem. Consumer–resource interactions can arise among a species pair when the first species produces a resource, and the second species consumes that resource (figure 2). This does not necessarily mean that whole organisms of the first species need to be consumed (as in typical predator–prey interactions), the resource produced by the first species may be just a part of the organism (as in herbivory), or may be a substance that an organism excretes (such as nectar excreted by plants that is used by nectarivores, sugar excreted by aphids that is used by ants or mucus produced in the digestive tract of a herbivore that is consumed by parasitic worms; figure 2).

Also, and importantly, the regular metabolic excretion products of species in ecosystems are generally resources to other species. Heterotrophic bacteria and fungi produce resources (mineral nutrients) for plants through metabolic excretion. Plants produce resources (coarse detritus) for earthworms, which produce resources (fine detritus) for bacteria, which produce again resources for plants. Plants produce resources for herbivores, which produce resources (dung) for dung beetles, which produce resources for bacteria, which produce resources for plants. Such recycling loops can lead to indirect mutualisms on the ecosystem level, which ‘draw’ additional resources towards them, increasing the productivity of all participants (Ulanowicz 1997). Even the external body surface of an organism can be an important limiting resource class (space) that it provides to other species (and will be competed for), as is the case for periphyton growing on aquatic macroalgae and macrophytes (in this case often with a negative net return through light interception by the periphyton) or epiphytes on the bark of a tree. In soft-bottom intertidal habitats with unstable sediments, the stable shells of bivalves form an important resource for macroalgae and other sedentary organisms that need solid ground. Similarly, the provision of nesting space for birds, and water and substrate for lichens by trees can be ranked under resource provision of the trees to other species. When studying interaction webs, it is important to separate such resources from the organisms that produce them, because multiple species will often contribute to the same resource (figure 2), while guilds of species compete for them. Such separation of resources and the species that produce them promote an integration between approaches from systems ecology (with focus on the dynamics of the resource compartments) and community ecology (with focus on the diversity of the organisms that produce them; figure 2).

We realize that species that are important in providing resources to several other species have been previously labelled as ‘ecosystem engineers’ by Jones *et al*. (1994), a concept that is becoming widely adopted (Wright & Jones 2006). However, strict application of this definition would classify virtually all species in most food webs as ecosystem engineers (including, e.g. all soil bacteria)—which is not what these authors intended. We think instead that the term ‘ecosystem engineer’ can much better be reserved for those species that strongly modify non-resource abiotic conditions (figure 1), resulting in all kinds of direct and indirect consequences for other species that are affected by these conditions. Such indirect effects may also include effects through changed resource availability, something that we will discuss later. Here we conclude that the full network of consumer–resource interactions in ecosystems generally will encompass more species than food webs, as the latter only deals with the subset of predator–prey interactions. And also, most food web studies and models ignore the indirect interactions among species that result from their differential production of resources through detritus production and excretion (e.g. Cohen *et al*. 1993*a*; Moore *et al*. 1993; de Ruiter *et al*. 1995; Neutel *et al*. 2002, 2007; Montoya *et al*. 2006).

The mortality and excretion of detritus and mineral nutrients by organisms yield a critical ‘downward’ producer–resource interaction (figure 1) between higher trophic levels and lower trophic levels (autotrophs, detrivores), which is required to close nutrient cycles and provide energy towards the detritus-based channel of food webs (figure 1). Organisms can show large difference in the amount and type of detritus they produce. For example, plants show large differences in the C/N ratio and lignin content of their litter, affecting the food basis of microbial detrivores, and thus the decomposition rate of detritus and hence nutrient recycling (Berendse *et al*. 1987). The consequences of this indirect interaction for community structure and ecosystem functioning are wide ranging, e.g. with respect to understanding the effects of climate change (Aerts 2006; Cornelissen *et al*. 2007). Further on in this paper we will discuss the consequences of these differences for the organization of consumer–resource interaction webs.

### (b) Non-trophic direct interactions

In addition to eating one another, species can show direct interactions in different ways (figure 1). Such non-trophic direct interactions become increasingly recognized. For example, the changes in physiological stress, behaviour (Bakker *et al*. 2005) or morphology (Werner & Peacor 2003) in prey caused by predation risk can substantially influence the net energy intake rate of the prey, and hence the attenuation of energy flow to higher trophic levels in ecosystems (Odum 1985; Trussell *et al*. 2006). Such changes may become ‘hard wired’ during the course of evolution, which means that predator-avoiding behaviour will be displayed even in predation-free situations (Brown 1999). Also, the ability of prey to defend themselves against predation can be induced by the presence of predators, as seen in some plant species that make more secondary compounds when subject to herbivory (Karban & Baldwin 1997). And, predators may adjust their phenotype in order to be able to handle different types of prey (Piersma & Lindstrom 1997; Piersma & Drent 2003; van Gils *et al*. 2006). In some plant species, herbivory induces the plant to produce chemical volatiles that attract the enemies of its enemies (Stowe *et al*. 1995). Also, the direct behavioural interference between organisms of a single or of different species (e.g. among large terrestrial predators) belongs in this category of non-trophic direct interactions (Menge 1995; Vahl *et al*. 2007), which can be uni- or bidirectional.

### (c) Interaction of organisms with environmental conditions

#### (i) Response to environmental conditions by organisms

Conditions are all things outside an organism that affect it but, in contrast to resources, are not consumed by it (Begon *et al*. 1990). Species at all trophic levels generally respond much more similarly to variations in environmental conditions (or stress) such as temperature than to resources. Over the last decades, the field of ecophysiology has gained strong insights into the physiological and morphological adaptations that allow species to cope with unfavourable environmental conditions, in both plants (Fitter & Hay 2001) and animals (Karasov & Martinez del Rio 2007). In addition, the field of behavioural ecology (Krebs & Davies 1997) has provided key insights into the origin and function of behavioural adaptations in response to unfavourable conditions. As a simple principle, all species that persist in an ecosystem can be assumed to have the appropriate physiological, morphological and behavioural adaptations to cope with the prevailing environmental conditions. However, not only the average conditions are important. Where short periods of resource shortage can be overcome by internal storage by organisms, short events of extreme conditions (very cold, hot, saline or anoxic conditions) can be fatal for organisms that lack the appropriate adaptations to cope with, or escape from those, and are therefore important for understanding community structure.

As the key physiological challenges posed by unfavourable conditions are generally the same for all organisms from microbes to plants to animals, this allows generalization of effects across widely different species. For example, lower temperature slows down the biochemical reactions of energy metabolism, reducing the available energy for resource uptake, growth and reproduction. As a result, the slope of the response to temperature of the rate of metabolism, development and growth of species of widely different taxonomic and trophic status (microbes, plants and animals) seems similar, which may be explained by the biochemical similarity of their basic metabolic pathways reflecting a common evolutionary origin (Gillooly *et al*. 2001, 2002; Brown *et al*. 2004; Savage *et al*. 2004). Such general knowledge on the temperature response of growth rate can be used to incorporate temperature effects on food web structure, e.g. to infer the balance between endotherms and ectotherms (Vasseur & McCann 2005).

#### (ii) Modification of environmental conditions by organisms

If species would respond only to the average environmental conditions, one may argue that such conditions are again not very relevant for understanding interaction webs. All species that occur in an ecosystem may simply be expected to have evolved adaptations to the prevailing conditions, which are external forcing factors to the local system. However, evidence is accumulating that many species can also strongly modify environmental conditions (Jones *et al*. 1994, 1997; Gutierrez & Jones 2006; Wright & Jones 2006), which introduces the potential of indirect species interactions through conditions, making them relevant to understanding the structure of interaction webs. Owing to physical and biochemical interactions, modification of conditions can change resource availabilities and have effects on autotrophs through two separate pathways (figure 1). For example, some European heathland plant species strongly lower the soil pH through their litter, which lowers the availability of phosphate in the soil for other plants, but also releases Al^3+^ cations in the soil solution, which are toxic for many other plant species and soil biota (Pegtel 1986). Also, *Sphagnum* mosses make the environment unsuitable for other (especially higher) plants through the same mechanism. These are exceptions; however, the general pattern seems that plants change abiotic soil conditions as pH and texture to their own benefit (van Breemen 1993).

The study of feedback effects of organisms on abiotic conditions has really taken off with the introduction of the concept of ecosystems engineers (Jones *et al*. 1994; Lawton 1994). More than a decade of research on this subject has now resulted in many examples of strong species–environment feedbacks in almost every habitat and ecosystem (Wright & Jones 2006), and has explored its evolutionary implications for niche construction (Odling-Smee *et al*. 2003) making it now time to start expanding food web theory with species–environment feedbacks. This is not an easy subject: species–environment feedback in a multi-species context, in which several species simultaneously respond to resources and conditions as well as affecting them, has been suggested to introduce strong nonlinearities in community and ecosystem dynamics, such as the emergence of multiple stable states, sudden regime shifts and chaos (Huisman & Weissing 1999; van de Koppel *et al*. 2001; 2005*b*; Scheffer & Carpenter 2003; Rietkerk *et al*. 2004; Carpenter *et al*. 2008). However, recent progress has been made with both implicit and explicit approaches for bringing non-resource environmental factors into interaction web theory.

### (d) Spatial interactions

#### (i) Colonization and immigration

Inspired by the theory of island biogeography (MacArthur & Wilson 1967), it is increasingly recognized that the dynamics and diversity of natural communities can only be understood well if immigration of new individuals or species from outside the system is taken into account (Caswell 1976; Hanski & Gilpin 1997; Hubbell 2001; Leibold *et al*. 2004). Even if a species does not meet the conditions locally required for long-term persistence, it may still persist due to immigration from a sink population. Also, when ecological drift or catastrophic events drive species locally to extinction, recolonization is required for continued persistence. Differences in dispersal strategy among species are therefore a key component in understanding community and food web structure (Levin *et al*. 2003). For example, limits to new species immigration are increasingly recognized as a limiting factor in the restoration of plant communities from which species have been lost (Bakker & Berendse 1999). The inability of particular species to reach a local community from the regional pool can be seen as a ‘filter’ that restricts the possible local species set (Ricklefs & Schluter 1993). The interplay of dispersal limitation with resource competition in determining community structure is increasingly explored within trophic levels (Leibold *et al*. 2004), but the consequences of dispersal limitation in a multitrophic food web context is still poorly explored.

#### (ii) Dispersal and harvesting

The human harvesting or exploitation of a particular population can be viewed as a spatial interaction that is equivalent to dispersal, as it removes individuals from the local ecosystem without direct population effects on the consumer (at least not on the same spatial scale). Therefore, harvesting strategies that remove individuals that would otherwise disperse to sink habitats have been proposed to be sustainable in the long-term for terrestrial ecosystems dominated by large herbivores (Owen-Smith 1988). Despite the development of elaborate harvesting models for population management (Ludwig *et al*. 1993; Hilborn *et al*. 1995), marine fisheries are increasingly leading to collapses of populations, especially at higher trophic levels in food webs (Pauly *et al*. 1998; Myers & Worm 2003; Berkes *et al*. 2006). In our final conclusions on conservation implications, we will discuss what we think is wrong here: we think that other-than-trophic interactions interfere.

#### (iii) Imports and exports of abiotic resources and energy

Energy and nutrients can enter ecosystems both in the detritus compartment (e.g. on the ocean floor or seashore) or in the abiotic resources compartment (e.g. eutrophication of mineral nutrients added by rivers to coastal marine systems). Especially in lake ecosystems, the consequences of added nutrients for trophic dynamics have been explored, with regard to trophic cascades and multiple stable states (Carpenter & Kitchell 1993; Scheffer & Carpenter 2003; Carpenter *et al*. 2008). The consequences of eutrophication for the food web structure of terrestrial ecosystems, e.g. through atmospheric nitrogen deposition, are much less documented. In a way, the effects of imports of abiotic resources and energy on food web structure may be easier to understand than the effects of modified environmental conditions, as the former affect food web structure only from the bottom-up, while the latter affect all trophic levels (figure 1).

While exports of energy and nutrients from ecosystems were not considered to be very interesting for a long time in community ecology (they were just ‘lost’), this has changed recently. Starting with the pioneering work of Gary Polis (Polis & Hurd 1996; Polis *et al*. 1997), food web ecologists increasingly realize that resource dynamics is not only governed by internal recycling of resources, but also in many ecosystems through spatial subsidies, leading to functional couplings between food webs in adjacent ecosystems (Huxel & McCann 1998; McCann *et al*. 2005). So the exports from one ecosystem may be required to understand the imports of other ecosystems, and hence their dynamics. This has led to the formulation of the concept of meta-ecosystems, which emphasizes the importance of spatial interactions among adjacent ecosystems through movement of propagules, organisms, energy and materials across system boundaries (Leibold *et al*. 2004).

### (e) Ecological relevance of physical and chemical interactions in ecosystems

Abiotic conditions such as soil or water salinity, soil or aquatic sediment texture, and soil, sediment or water pH and redox highly affect the availability of resources to organisms (Schlesinger 1991). Such geochemical interactions can therefore play a key role in the structure and functioning of ecosystems, both on short (ecological) and long (geological) time scales. For example, the texture (relative contribution of sand, silt and clay) of marine sediments strongly affects its aeration, and oxygen is an important resource for many species of benthic infauna. Also, soil and sediment aeration affects many geochemical reactions through its impact on redox potential. Both are also subject to organismal feedbacks, through bioturbation (affecting aeration and texture) and filter-feeding (affecting texture) (Herman *et al*. 1999; Widdows *et al*. 2004). For terrestrial ecosystems, fire should be mentioned here as special kind of physical interaction that is important as it can lead to rapid loss of energy and some nutrients (such as nitrogen) from the detritus compartment through volatilization, suddenly moving nutrients from coarse detritus to the abiotic resources compartment (such as phosphorus), short-cutting the decomposition chain from detritus to mineral nutrients (McNaughton *et al*. 1998). Also, fire leads of course to temperature conditions lethal for many plants and animals (unless they have adaptations to cope or escape those extreme conditions).

### (f) Environmental forcing

In addition to the biotic influences it receives, local abiotic conditions are also often subject to strong external forcing (figure 1), for example when regional climatic conditions affect local air, water or soil temperature, without receiving much feedback from it. This external forcing is the key ‘point of entry’ in studying not only the effect of climate change on food webs, but also how toxic pollutants will affect trophic structure and ecosystem functioning. Surprisingly, despite the existence of good indicators for its operation, e.g. in the level of synchrony between species in long-term ecological monitoring (Bakker *et al*. 1996), environmental forcing has hardly received any attention in the study of consumer–resource interactions, food webs or other interaction webs (Vasseur & McCann 2005; Vasseur & Fox 2007; Loreau & de Mazancourt 2008).

## 4. Two main axes of food web organization

The six main types of ecological interaction outlined in §3 can be used to map (parallel) ecological networks in different ecosystems in a similar, standardized way. Before exploring this idea further, however, we first return to the first interaction type (consumer–resource interactions) to expand upon the classic axis of food web organization (vertical trophic position) with a second, horizontal axis. This second axis will facilitate the development of a testable template on the basis of which food webs can be compared, to apply both to a number of different ecosystems as ‘proof of concept’, in combination with the previously listed six main interaction types.

A strong point of food web ecology is its promise for generality: it holds the potential to be useful in comparing very different ecosystems, and hence produce general conclusions on the organizational forces and principles at work. However, this ability to compare is currently hampered by our inability to assign species generic functional roles. Yet, such system-independent roles of species are of great fundamental and applied interest. This role should characterize the general topological position and functional importance of a species in ecological networks, independent from the particular web under study. Current functional classifications mainly use the trophic position, as top predators (Finke & Denno 2005; Scheffer *et al*. 2005; Borrvall & Ebenman 2006), mesopredators (Elmhagen & Rushton 2007), herbivores and primary producers. For interaction webs including species–environment interactions, the importance of ecosystem engineers has been recognized for species that strongly modify abiotic conditions, and hence resources to other species (Jones *et al*. 1994; Lawton 1994; Wright & Jones 2006). But can other main axes of organization be identified? We suggest that a more structured approach is required for each of the six types of interaction that define generic species groups by their topological position, and hence their functional roles in ecosystems. The result would be an ‘interaction web template’ that should fit to describe any ecosystem.

**Figure 3:**
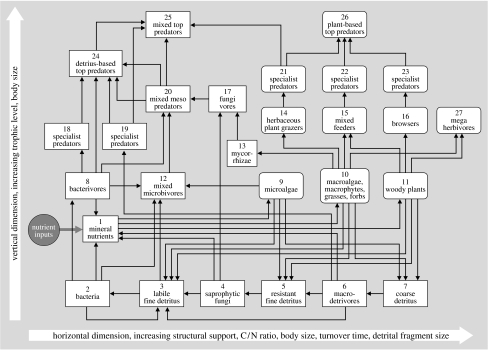
A proposed general functional group classification for food webs, intended for comparing food web structure between ecosystems. The compartments are linked by trophic interactions and detritus production (figure 1), the basic interactions that structure food webs. The compartments are arranged along two main axes of organization. The vertical axes reflects the approximate trophic position of species, arranged from low (bottom) to high (top), generally leading to an increase in body size. The horizontal axis reflects a stoichiometric axis, reflecting a larger size, coarser structure, higher carbon:nutrient ratios and slower turnover of the primary produces along the axis from left to right (increasing structural support). Associated with increasing body size is a greater ability of detrivores and herbivores to ingest and digest poorer and coarser quality food, and lower per mass energy and nutrient requirements of these organisms. As a result of the processes that change along both axes, the body size of organisms increases from bottom left to top right in the scheme. See figure 1 for the interpretation of the different types of arrow.

For consumer–resource interactions, we propose such a template in figure 3 to explore the usefulness of this idea. Each numbered box is a functional group, generally consisting of a group of species that is competing for resources that they obtain from one or more other functional groups in the web (figure 2). This general web is ‘anchored’ at the bottom left, where algae and other autotrophs produce biomass, and heterotrophic bacteria decompose the detritus produced by plants and higher trophic levels, both at a high rate of turnover. We suggest that, starting from here, consumer–resource interaction webs are organized along two main axes. The vertical axis is the classic trophic position axes, forming food chains of species towards increasingly higher trophic levels. Generally, the size of species increases along the vertical axis, as predators generally need to be bigger than their prey to hunt and handle them efficiently (with the exception of pathogens, which we discuss later) (Cohen *et al*. 1993*b*; Brose *et al*. 2006). We suggest a new second major axis of food web organization: a stoichiometric axis. At the lowest trophic levels, this axis is driven by two main evolutionary radiations: (i) the competitive struggle for light between plants (as is still observable during primary succession, changing the dominance by algae, to herbaceous plants, to trees), leading to the formation of plants with more and more structural support (cellulose, lignin, etc.) in an effort to overtop each other, and (ii) a radiation of detrivores other than bacteria, which could physically fragment (macrodetrivores) and biochemically decompose (fungi) the coarser, poor-quality plant material that these taller, mechanically better supported plants increasingly produced. Therefore, the horizontal axis is a stoichiometric axis (Sterner & Elser 2002), representing a decreasing C/N ratio of the plant material produced and a lower turnover rate of its compartments. Within the next herbivore trophic level, this horizontal axis has also resulted in size radiation of consumers, not driven, however, by the need to be bigger than their prey, but by the need to be able to digest it. Bigger herbivores can handle poorer quality food due to the longer residence time of food in their stomach, and lower per mass energy requirement, leading to a more favourable ratio of digestive capacity to metabolic requirement (Demment & van Soest 1985). The resulting increase in herbivore size from the need to handle poorer quality (niche-based species radiation) may then have triggered an evolutionary arms race between herbivores and predators, causing a size increase in predators as well (Owen-Smith & Mills 2008*a*) and also resulting in some herbivores eventually ‘escaping’ their predators by growing too big, so-called megaherbivores (Owen-Smith 1988). The two independent axes of food web organization suggested in figure 3 therefore cause strong body size variation to exist both within and across trophic levels, with the smallest species found at the bottom left, and the largest species at top right. The organization of food webs are a testable hypothesis, which requires the compilation of data on both quantitative trophic position (e.g. through stable N isotopes) and stoichiometric position (e.g. through measuring C/N or C/P ratios of diets and excretion products, turnover rates), facilitating a quantitative comparison of the resulting patterns across ecosystems.

In the remaining of this paper, we will qualitatively explore this ‘food web template’ (figure 3), together with the six main interaction types we identified (figure 1), for a number of very different food webs for a first proof of concept. For each of three ecosystems, we will explore the interaction web based on both consumer–resource interactions, as well as on the other types of interaction shown in figure 1. In the latter case we focus especially on the interaction between species and biotic (non-resource) factors.

## 5. Parallel interaction webs: case studies

### (a) European intertidal mudflats

**Figure 4:**
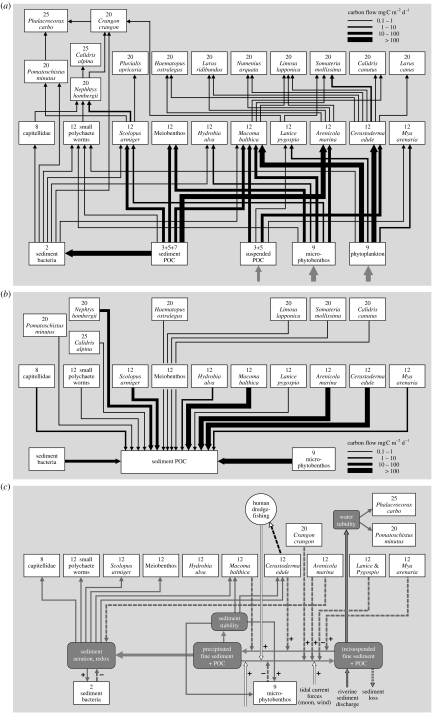
The general framework for studying ecological networks (figures 1 and 2), as applied to the soft-bottom intertidal mudflat ecosystem of the Wadden Sea (Sylt-Rømø part, Denmark). This ecosystem consists of several subwebs. The interactions for the ‘*Arenicola* flats’ subweb are presented here. (*a*) The interaction web based on consumer–resource interactions (food web), where the topology of the web and the weight of the interactions (presented here as carbon flow, in mgC m^2^ d^−1^) is based on measured fluxes as presented by Baird *et al*. (2007). (*b*) The interaction web for the same ecosystem drawn for the detritus production part of consumer–resource interactions, based on measured fluxes as presented by Baird *et al*. (2007). (*c*) The inferred interaction web for the same ecosystem for other than consumer–resource interactions, drawn for important effects of species on abiotic conditions (ecosystem engineering), response of species to abiotic conditions, external forcing, material inputs and losses, and various physical and chemical interactions, based on information from various sources (Whitlatch 1981; Flach 1992; Herman *et al*. 1999; Widdows *et al*. 2004; Coco *et al*. 2006; Huxham *et al*. 2006; Lumborg *et al*. 2006). The key interaction in this web is the effect of organisms on physical conditions. Specifically, the web outlines the influence of organisms on the sedimentation rate of fine sediment versus its resuspension, where some biota promote the sedimentation, while others promote or inhibits its resuspension. Interaction weights were not available for the interaction web shown in (*c*). Numbers inside each box indicate the trophic functional group (figure 2). See figure 1 for the interpretation of the different types of arrow.

The first web consists of the food web formed by marine plankton, benthic invertebrates and their predators on the Sylt-Rømø soft-bottom intertidal flats in Denmark (figure 4), based on the data in Baird *et al*. (2007). We observe that the food web already at the herbivore level is firmly based on both the detritus and herbivore channels (figure 1), as most benthic organisms feed on both. Primary production in the system arises from two groups of autotrophs: the pelagic microalgae with fast turnover and the microphytobenthos (diatom mats) growing on top of the sediment with slower turnover. The web is dominated by a layer of mixed microbivores (the benthos) that feed on both detritus and microalgae, and are fed upon in turn by mostly a single layer of mixed mesopredators (the birds). The mixed microbivores (mostly worms and bivalves in this case) are especially important in producing detritus that enters the pool of sediment particulate organic matter (figure 4*b*), hence producing resources for other microbivores, and mineralizing nutrients for the microalgae. The horizontal, stoichiometric axis of organization can also be clearly recognized. A strong size differentiation among the mixed microbivores exists, in which bigger species such as the sediment-feeding worm *Arenicola* probably deal with particle sizes that cannot be handled by the much smaller meiobenthos (such as nematodes), but produce detritus than that can be used by smaller mixed microbivores. Also, a megaherbivore is found in the system, as the bivalve *Mya arenaria* becomes so big (and lives so deep) that halfway through life it becomes effectively predation free (Zwarts & Wanink 1984; Zaklan & Ydenberg 1997)—thus representing an ‘elephant of the mudflats’. On the left side of the web, where species become smaller and smaller, and where the web is based on more finer sized detritus particles, the number of trophic levels increases, facilitating mixed top predators, and higher trophic levels that can persist on the ‘high-quality’ end of the stoichiometric axis. We suggest that this triangular structure is a general pattern across both marine and terrestrial ecosystems.

Figure 4*c* shows the interaction web for the same ecosystem, but now drawn not for classic consumer–resource interactions, but for a set of other relevant interactions out of our list of six. This interaction web is drawn from information on a variety of sources (Reise 1985; Herman *et al*. 1999, 2001; Piersma *et al*. 2001; Widdows *et al*. 2004; van Gils *et al*. 2006; van Oevelen *et al*. 2006). Important abiotic (non-resource) conditions in this system are the texture and stability of the sediment, the aeration or redox of the sediment and the turbidity of the water. These abiotic conditions are mutually dependent on each other through physical and chemical interactions (figure 4*c*). A central abiotic process is the balance between sedimentation of fine sediment and its resuspension due to the turbulence of the upcoming and outgoing tide (the mudflats are flooded twice a day by sea water from the tidal gulleys). If more fine sediment (a mixture of organic matter, silt and clay) goes into suspension than on average settles, then the mudflat becomes more sandy, as the fine sediment is exported from the system by tidal currents. Also, high resuspension rates increase the turbidity of the water. If more fine sediment settles than goes into resuspension, the mudflat becomes more muddy. This balance between settlement and resuspension is affected by a mixture of biotic and abiotic processes, some internal to the system, some externally imposed. Settlement of fine sediment is promoted by filter-feeding bivalves, which filter it out of the water and deposit it in their neighbourhood as pseudofaeces. Resuspension of the sediment is promoted by the digging (bioturbation) activities of lugworms (*Arenicola*) and the foraging of shrimp (*Crangon*). Also, human dredging for edible cockles (*Cerastoderma*) not only has depleted their stocks, but also has promoted the resuspension of fine sediment, hence promoting sediment loss and sediment instability. On the other hand, microphytobenthos and sand mason worms (*Lanice*) ‘glue’ the sediment together, hence reducing resuspension.

The aeration (associated with redox) of the sediment highly depends on the texture (better aeration in coarser sediments) and hence on the balance of settlement–resuspension of fine material. Organisms not only highly affect the abiotic condition of sediment texture, aeration and water turbidity, but they also strongly respond to it. Better aeration promotes decomposition by aerobic heterotrophic bacteria, and promotes most species of smaller benthos. High sediment stability seems required for the establishment after spatfall (recruitment) of the bivalves. Higher water turbidity reduces the foraging success of fishes and birds that hunt by sight, hence relaxing top-down forces in the system (figure 4*c*). Several potential feedback loops exist in the network of figure 4*c*. For example, microphytobenthos promote their own growing conditions by stabilizing the sediment, while filter-feeders make conditions less suitable for their own recruitment, by decreasing sediment stability, which may lead to population cycles. The overall picture that arises from figure 4 is that the network of consumer–resource interactions will be highly affected by the network of other interactions that operate in parallel, and vice versa. Trophic interactions can ramificate into the abiotic network, and non-trophic interactions will ramificate into the consumer–resource network. Neither network seems to have clear priority over the other in determining the abundances of species and the functioning of the ecosystem.

### (b) North American short grass prairie

**Figure 5:**
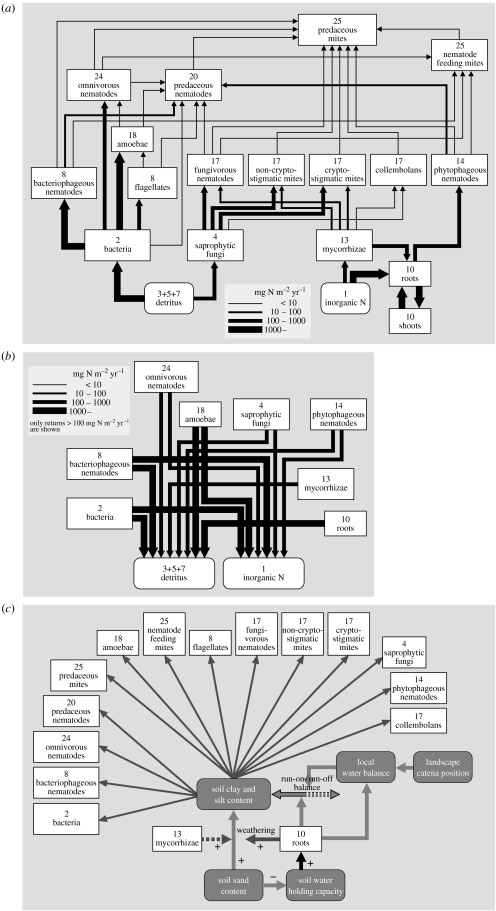
The general framework for studying ecological networks (figures 1 and 2), as applied to the soil subweb of the short-grass plains ecosystem of Colorado, USA (Central Plains Experimental Range). (*a*) The interaction web based on consumer–resource interactions (food web), where the interaction topology and weights (presented here as nitrogen flow, in mgN m^2^ yr^−1^) is based on measured and calculated fluxes as presented by Hunt *et al*. (1987). (*b*) The detritus-production part of the consumer–resource interaction web (in mgN m^2^ yr^−1^), based on measured and calculated fluxes as presented by Hunt *et al*. (1987). Only returns greater than 100 mg N m^2^ yr^−1^ are shown. (*c*) The interaction web for the same ecosystem based on species effects on abiotic conditions and species responses to abiotic conditions, as inferred from information provided for this ecosystem by Hook & Burke (2000), and general information for other drylands (van Breemen 1993; Austin *et al*. 2004). The key process here is the modification of soil texture by plants and fungi (through effects on weathering and run-on/run-off balance, and the high sensitivity of soil biota to texture. Interaction weights were not available for the interaction web shown in (*c*). Numbers inside each box indicate the trophic functional group (figure 2). See figure 1 for the interpretation of the different types of arrow.

The second web that we analysed is the soil food web of the short grass prairie, Colorado, North America. Hunt *et al*. (1987) measured and estimated the flows of nitrogen between different functional groups of soil biota in this system (figure 5*a*). This web is a subweb from the larger consumer–resource network of this ecosystem, as it deals only with below-ground trophic interactions. Although composed of very different species, we see that the general structure resembles the intertidal food web, with a more complex, reticulate structure on the left (‘small—high resource quality–fast turnover’) side of the web, while being ‘flatter’ for the right side (‘large—lower resource quality–slower turnover’) of the web. The basis of the web is formed by two energy channels: one detritus based and the other plant based, the latter which splits into a bacterial and fungal channel. Rooney *et al*. (2006, 2008) have suggested that the coexistence of such channels with a different flux and turnover rate contribute to the stability of food webs to external perturbations. Towards higher trophic levels, all channels merge again due to the presence of omnivorous top predators, but stay longer separate than in the intertidal mudflat example. On the bacterial-based side of the web, weak intraguild predation is found by omnivorous nematodes on amoeba, and by amoeba on flagellates, while all groups also feed on bacteria. This creates consumer–resource feedback loops that have been suggested to contribute to the stability of the entire web (Neutel *et al*. 2002). Similar loops of intraguild predation may arise in the macrofauna of the litter layer (beetles, spiders, etc.); however, these groups were not samples in the study of Hunt *et al*. An important difference to the marine food web of figure 4 is the central role played in this web by fungi and fungivores, pointing at the poorer C/N ratio of the organic matter produced by the plants in this ecosystem. Similar to the marine web, all lower trophic levels make important contributions to the detritus and mineral nutrient compartment (figure 5*b*), introducing important producer–resource interactions between species at lower trophic levels. It should be noted that the researchers in this case aggregated species in functional groups, each incorporating up to tens to hundreds of species that compete for resources. So where the soil food web seems to be more strongly structured by predator–prey interactions, while the intertidal food web seems structured more by competition, this may be an artefact of the differential level of aggregation chosen.

Figure 5*c* shows a parallel network of non-trophic interactions that operates in this system simultaneously, based mainly on information provided by Hook & Burke (2000). The central abiotic process here is the dynamics of soil texture (similar to the intertidal ecosystem, but with slower dynamics), where the soil silt and clay content (fine fraction) are determined by inputs through weathering (promoted by plants and mycorrhizae) and by the run-on/run-off balance of silt and clay, which depend on the catena position in the landscape and on the vegetation cover. Soil texture has a direct effect on most soil biota through affecting their ability to move, especially in combination with the soil water content (not shown), which depends on texture, run-on/run-off balance (determined by catena position) and evapotranspiration (determined by rainfall, vegetation cover and radiation). Soil water availability especially has a strong impact on bacterial and fungal decomposition, and hence the rest of the food web that is based on these groups (figure 5*a*). The non-trophic network contains various feedbacks, e.g. where plants promote texture to their own benefit (van Breemen 1993). Again, both the consumer–resource network (figure 5*a*,*b*) and the non-trophic ecological network (figure 5*c*) highly intertwine, where change in one network can ramificate or can be amplified or inhibited through the other network, and vice versa.

### (c) African savannah

**Figure 6:**
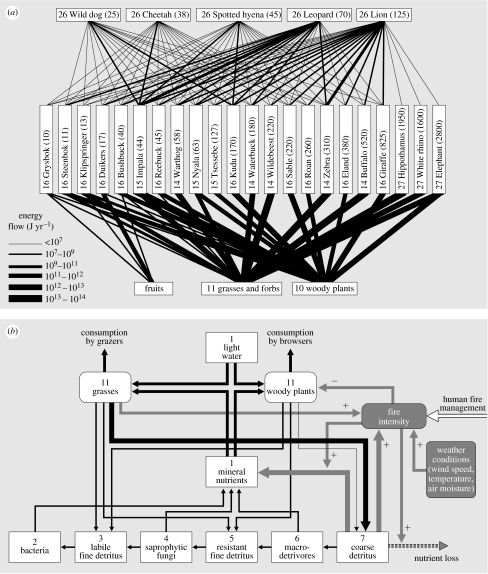
The general framework for studying ecological networks (figures 1 and 2), as applied to the savannah ecosystem of Kruger National Park, South Africa. (*a*) The subweb of consumer–resource interactions that involves larger mammalian herbivores and their predators. The interaction topology and weights (presented here as annual energy flow, in J yr^−1^) for the herbivore–predator interactions is from the data presented by Owen-Smith & Mills (2008*a*), where feeding rates based on meat were converted to energy flows, using a conversion of 1 kg meat=23 600 J (Karasov & Martinez del Rio 2007). The energy flow (J d^−1^) between all plants and each herbivore population was first calculated allometrically as *N*×7940 *W*^0.646^ (Demment & van Soest 1985), where *W* is the body mass of the herbivore (g) and *N* is the population density, as reported by Owen-Smith & Mills (2008*a*). Then, this total energy flow per herbivore species was partitioned over its three main food item classes according to the proportional diets given by Gagnon & Chew (2000). (*b*) The interaction web for the same ecosystem based on physical and chemical interactions, detritus-based consumer–resource interactions, and interactions between organisms and abiotic (non-resource) conditions. The key process here is the role of fire, short-cutting nutrients away from the horizontal decomposition pathway (figure 2), making nutrients partly directly available to plants through burning off energy and carbon, while partly stimulating nutrient losses through ash run-off and gaseous losses. Also, fire kills (especially young) trees, while grasses are much more resistant to fire (Bond & Vanwilgen 1996). The higher coarse detritus production by grasses compared with trees increases the fuel loads, which promotes fires, benefiting grasses in competition with trees for light and water. On the other hand, if trees manage to outshade grasses during long fire intervals, then the fuel load is highly reduced, and fires become permanently suppressed. Also, high grazer densities can deplete grass biomass, which suppresses fires, and can lead to tree invasion (Dublin 1995; Sinclair 1995). This makes the outcome of the tree–grass interaction in grazed tropical systems at intermediate rainfall in the presence of fire highly unpredictable (Bond 2005), but very diverse in large herbivores (Olff *et al*. 2002). Quantitative interaction weights were not available for the interaction web shown in (*b*). Numbers inside each box indicate the trophic functional group (figure 2). See figure 1 for the interpretation of the different types of arrow.

Our last interaction web analysed in this way is that among plants, large herbivores and their predators as found in the Kruger National Park savannah ecosystem in South Africa (figure 6). The energy flow in the trophic part for the herbivore–predator web part was calculated from the data recently published by Owen-Smith & Mills (2008*a*), while the plant–herbivore part was calculated using diet data provided by Gagnon & Chew (2000), and using densities and allometric equations. It should be noted first that the web shown in figure 6*a* is again a subweb of the total consumer–resource network found in this ecosystems. For example, small mammals and invertebrate herbivores (such as grasshoppers) were not included, nor was the entire detritus-based decomposition chain of the food web. Clearly, this consumer–resource subweb is firmly rooted in two energy channels formed by the grasses (fast turnover) and woody plants (slow turnover). Within the herbivore trophic level, a strong stoichiometric body size gradient is found, where species seem to alternate within each size class between the two energy channels. It is unclear yet whether this is a general principle. Resource partitioning between herbivores of different size is a classic theme of investigation in tropical savannahs (Vesey-Fitzgerald 1960; Bell 1971). The outcome of earlier studies is that body size differences promote coexistence along gradients of productivity and plant quality, with bigger species being better able to handle poorer quality, but needing more food (Prins & Olff 1999; Ritchie & Olff 1999; Haskell *et al*. 2002; Olff *et al*. 2002). This provides coexistence opportunities, especially in the presence of spatial heterogeneity of food quality and quantity (Ritchie & Olff 1999; Cromsigt & Olff 2006; Cromsigt & Olff 2008), and leads to facilitation interactions (Vesey-Fitzgerald 1960; Prins & Olff 1999; Arsenault & Owen-Smith 2002). The ‘horizontal’ size spectrum of herbivores forms again a niche axis along which competing predators partition their prey (figure 6*a*), with generally bigger herbivores sustaining bigger predators, but with strong niche overlap. Also in this case, spatial heterogeneity is expected to contribute to the coexistence of competing predators, and are important indirect effects observed (not shown in the figure) of vegetation structure on predator–prey interactions (Hopcraft *et al*. 2005). Although from a trophic perspective, this web perfectly forms three layers, this is caused by the choice of the researchers to include only direct predator–prey interactions. From large to small, predators have been observed to form a competitive hierarchy, where predators interfere through kleptoparasitism and behavioural interference (chasing each other away from their kills and territories). Inclusion of such effects would bring a more ‘vertical’ structure in the interaction web. Also, it should be noted that the observed prey choices of these savannah predators are quite flexible due to adaptive foraging (Owen-Smith & Mills 2008*b*), emphasizing again the importance of ‘conditional probabilities’.

Figure 6*b* shows the web of key non-trophic interactions which we think operate in this ecosystem, in combination with its important trophic links. The main abiotic variable is the intensity of savannah fires that regularly occur, which depends on weather conditions, fuel load formed by coarse detritus (mostly formed by grasses) and human fire management (when and where to light fires). In addition, the system holds a strong legacy of the past, as the fuel load depends on the duration since the last fire (a management decision). Intense fires have two main effects: (i) they kill woody plants (especially young ones) and (ii) they transfer nutrients in coarse detritus partially into the mineral nutrient pool, while partially facilitating nutrient loss through combustion and run-off of ash. Fires therefore provide a short cut, temporally shutting down the decomposition chain that the coarse grass detritus would enter if the system was not burned. This also locks out all higher trophic levels that could be based on this detrital chain (compare with figure 5*a*). From an ecosystem perspective, fire should therefore not be viewed as a ‘non-selective herbivore’ (Bond & Keeley 2005). Instead, it operates as a very fast and efficient detrivore. Again, the consumer–resource network and the network based on other types of interaction strongly interact in this system. Through killing trees, intense fires promote the balance in competition for resources (water and nutrients) between trees and grasses in the advantage of the latter. However, if a site is not burned for a long time then trees outshade grasses and create moist microclimates, which may suppress fires for a long time. By the sudden mineralization of nutrients, fires promote nutrient uptake by grasses, from which herbivores profit. Also, the decreased woody cover and shorter grass that results from intense fires reduce the hunting success of their predators, providing a dual advantage. The net result of these processes is strong spatial and temporal unpredictability of ecosystem configurations in climatic regions where fires occur (Bond 2005).

We conclude from the examples in figures 4–6 that the application of a food web template as developed in figure 3 seems to really work, and facilitates the comparison of the role of functionally equivalent species across very different ecosystems. Also, for each of the ecosystem observed, various interaction webs can be drawn using the six main types of interaction shown in figure 1. These different interaction webs were found to show strong mutual interferences, which calls for their joint analysis.

## 6. Linking ecological networks with different types of interaction

**Figure 7:**
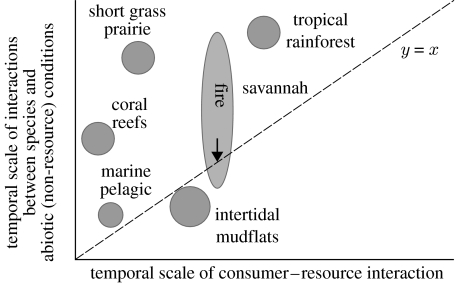
The positioning of various ecosystems in the phase space formed by the temporal scale of the consumer–resource interactions that structure them, and the temporal scale of the interactions between species and abiotic (non-resource) conditions. Along the *x*-axis, the growth rate of organisms decreases, while their body size and resource turnover times decrease. Along the *y*-axis, the rate of change of abiotic (non-resource) conditions decreases, such as microclimate, soil texture, physical structures created by organism (reefs and soil caliche layers) and mixing rate of the medium (water and soil).

So given webs based on different types of interaction occur in ecosystems, how do we link these different webs, conceptually and in models? We think that we are just beginning to understand this, and will suggest some directions. A first step in approaching this problem is to think about the temporal and spatial scales involved in each class of processes that forms separate interaction webs. The important question is then whether these scales are clearly separate or merge. Figure 7 shows a qualitative graph of the phase plane of the temporal scale of consumer–resource interactions (increasing with size and lifespan of organisms involved, and decreasing with turnover time of resources in biotic compartments), and the temporal scales of interactions between species and abiotic (non-resource) conditions (decreasing with the rate of change of key abiotic factors). We have shown the tentative position of different ecosystems in this phase plane, including the three we discussed in the previous section. For the short grass plains, one can argue that these temporal scales are clearly separated, where the landscape-forming processes that determine texture dynamics along landscape gradients are larger and slower than the consumer–resource interactions between the organisms in the food web. The same may hold, for example, on coral reefs where the reef-building process (deposition of calciferous structures) is a much slower one than the actual consumer–resource dynamics that govern it (filter-feeding anthozoans). In this case, a hierarchical approach can be used (Allen & Starr 1982), where the dynamics of the species–abiotic environment interactions is solved first, followed by solving the consumer–resource dynamics, under the assumption of quasi-steady state of the abiotic conditions. However, in the intertidal mudflat example, we saw that the time scales of the consumer–resource interactions started to blur with the time scales of the species–abiotic environment interactions, and the latter may even be faster than the first. The savannah example had a bit of both (figure 7). The special feature of fire in this system suddenly speeds up dynamics in environmental conditions to become much faster than consumer–resource interactions, but only temporarily. In the case where time scales of different types of process cannot be clearly separated any more, we suggest both implicit and explicit approaches to addressing the interplay between the parallel networks at work.

### (a) Implicit approaches for linking networks

Recently, Arditi *et al*. (2005) proposed an ‘interaction modification’ approach that implicitly deals with the modification of environmental conditions by organisms, a concept that was further developed by Dambacher & Ramos-Jiliberto (2007) and Goudard & Loreau (2008). In addition to the interaction of an organism with its own resources, organisms can also modify the interaction between other organisms and *their* resources. Arditi *et al*. propose to capture this through a ‘net effect’, without taking the explicit modification of the environmental conditions, and the response of species to them, explicitly into account. For example, microbial crusts in the desert can reduce the infiltration of water, which strongly affects the consumer–resource interaction of higher plants with soil water (West 1990). Or lugworms (*Arenicola*) in soft-bottom intertidal sediments strongly promote the aeration of the sediment through bioturbation, which facilitates many other larger detritus feeders that require such aerobic conditions (Herman *et al*. 1999). Trophic relationships higher up in the web can also be dealt with through this approach. For example, thorny shrubs or chemically defended plants can reduce the consumption by large herbivores of palatable tree saplings, which has large consequences for ecosystem dynamics and the formation of spatial structure in grazed ecosystems (Olff *et al*. 1999; Bakker *et al*. 2004; Smit *et al*. 2007, 2008). Also, the role of some organisms such as mussels and macroalgae in forming safe sites, where marine animals can find shelter against their predators, could be captured by this modelling approach.

### (b) Explicit approaches for linking networks

An alternative to the interaction modification approach is the explicit modelling of the modification of the environmental conditions and/or resources affected by the organism involved, with indirect consequences of other interactions. Examples of studies using this approach include the analysis of how plant cover in semi-arid areas affects the water infiltration capacity of the soil, and hence the soil water balance, which in turn affects plant–herbivore interactions (Rietkerk *et al*. 2000). The resulting scale-dependent feedbacks introduce interesting nonlinearities and sometimes catastrophic behaviour in the ecosystems involved (Rietkerk *et al*. 2004; van de Koppel *et al*. 2005*a*,*b*; Kefi *et al*. 2007). Therefore, such feedbacks can be viewed as destabilizing the system.

However, feedbacks of organisms on abiotic conditions also have the potential to stabilize ecosystems. Probably the most famous example of such a stabilizing feedback has been the ‘daisy world’ mini-model, originally presented by James Lovelock (Watson & Lovelock 1983) to illustrate mechanisms that could underlie his ‘Gaia hypothesis’, i.e. the importance of life in promoting homeostasis in the global atmospheric composition and climate, with returning benefits for this life (Margulis & Lovelock 1974; Lovelock & Watson 1982). Although highly controversial upon its presentation, the operation of vegetation–climate regulatory feedback is now generally accepted (e.g. through differences in the albedo of snow, different vegetation types and bare ground, the temperature changes resulting from that, and the response of snow and vegetation to such temperature change), an increasingly important component in current global change models (Luo 2007). Unfortunately, the original Gaia idea was poorly communicated with probably too much emphasis on ‘ultimate goals’ that the biosphere would have (Wilkinson 1999; Free & Barton 2007), which may explain why only very recently ecosystem-level regulatory feedbacks are receiving some serious theoretical scrutiny (Lenton 1998; Seto & Akag 2007; McDonald-Gibson *et al*. 2008; Wood *et al*. 2008), and also now in the context of food webs (Bagdassarian *et al*. 2007). The idea of the importance of such regulatory feedbacks fits well with our previous recognition of the importance of causes of organization that arise at a particular level of organization through a specific topological arrangement of interactions. Community and ecosystem ecologists could also benefit here from insights into biochemical networks, where ideas on the importance and operation of regulatory feedbacks are well established.

## 7. Concluding remarks

### (a) Beyond predator–prey interactions

In this overview, we have tried to outline a framework that may be useful in the further theoretical, observational and experimental studies of parallel ecological networks. We need more structured approaches that map for different ecosystems how strong ‘other-than-food-web’ interactions affect species and ecosystem dynamics. Also, we need to quantify on what spatial and temporal scales these processes operate, as this has consequences for modelling approaches. The strong emphasis for example in soil ecological network studies on trophic interactions does not mean that modification of the abiotic habitat by organisms, or dispersal, play a less important role. There may be just less of a tradition to investigate it. Instead, researchers in intertidal mudflat ecosystems have a strong tradition of studying interactions between organisms and abiotic (non-resource) conditions, and on studying imports and exports of organic matter, but that does not mean that food web interactions or dispersal are less important as structuring forces. Similarly, the strong emphasis in rainforest research on dispersal and immigration (e.g. Hubbell 2001) does not mean that trophic interactions, or interactions between plants and soil formation, are less important. Similarly, in savannah ecosystems, the dynamics of the detritus-based part of the food web (and its interplay with fire) seems largely ignored, while large herbivores and their predators have received most attention. Rather than *a priori* assume that a particular type of interaction is dominating the structure and functioning of a particular ecosystem, we need new ways to quantify the relative importance of different main types of interaction as shown in figure 1, and new approaches to study their interplay.

### (b) Keystone species or keystone interactions?

Ecologists recognized early on that not all species or all interactions are equally important for the structure of the communities and the functioning of the interaction webs that they form. Paine (1969) launched the influential concept of keystone species, referred to as species that preferentially consumed and held in check another species that would otherwise dominate the system, noting that such species may be unimportant as energy or material transformers. Broadening later to include also non-trophic interactions and emphasizing especially the importance of subordinate species with a role disproportional large to their abundance (Power *et al*. 1996), many examples of such species have now been documented (Boogert *et al*. 2006). Approaching this problem more from a systems perspective, Holling (1992) has suggested that only a small set of plant, animal and abiotic processes structure even the most diverse ecosystems. However, a problem with the current keystone species literature is that it seems to lack generality. Merely, it can be characterized as an increasingly long list of case studies with little general pattern.

Following Paine's and Holling's lead, we can use our proposed list of six major interaction types to ask whether a *particular type* of interaction has a very strong impact on the overall community structure, and seek for generality on that level. For example, the importance of predation on herbivores yielding keystone interactions has been widely demonstrated in rocky intertidal (Paine 1969; Menge *et al*. 1994), kelp forest ecosystems and freshwater lakes (Carpenter & Kitchell 1993). For tropical forests on the other hand, Hubbell (2001) has proposed that tree propagule immigration is the keystone interaction that dominates the community structure and dynamics. For soft-bottom intertidal ecosystems, the modification by organisms of abiotic conditions, which include sediment aeration, texture and hydrodynamics, has been viewed as a keystone interaction (Herman *et al*. 2001; Widdows *et al*. 2004; van Wesenbeeck *et al*. 2007) strongly structuring communities and affecting ecosystem functioning. For an understanding of the functioning of coastal desert communities, the energy and material inputs from the neighbouring sea ecosystem has been viewed as the keystone interaction (Polis & Hurd 1996). Making such an inventory to see which ecosystems are dominated by which kinds of interaction is a different approach than the quest for keystone species. For the tropical rainforest example, not one single species may dominate the community. Yet, the immigration and colonization seem key to understand its structure. This fits with the conclusion of Paine (1969) and later authors that keystone species are often widely different in traits and hard to predict. So where it may be predictable that consumer–resource interactions dominate in rocky intertidal ecosystem, it may be less well predictable which species pick up that role (e.g. depending on historic and geographical factors (Ricklefs & Schluter 1993)). We thus conclude that more generality can be found when exploring keystone interactions rather than keystone species.

### (c) Dealing with parasites and pathogens

**Figure 8:**
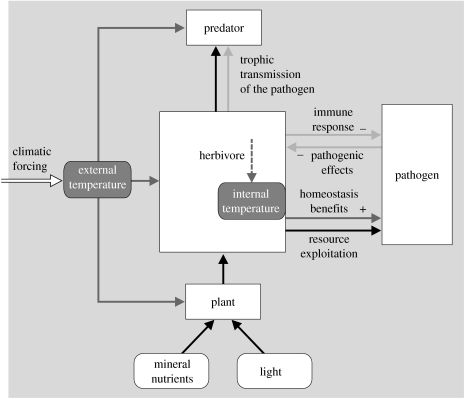
The role of pathogens in ecological networks, using the various interaction types outlined in figure 1. Shown is an example where a pathogen attacks a herbivore. Pathogens are successful not only because they use resources provided by hosts, but also especially because they profit from the internal, often homeostatic non-resource conditions (such as temperature, pH) that the host creates within its own body. Therefore, small, exothermic organisms such as bacteria and helminth worms can achieve high populations in situations where they otherwise would be regulated by external (e.g. climatic) forcing; they become uncoupled from those. In addition to receiving favourable conditions and resources from the host, the pathogen can impose direct non-trophic negative (e.g. toxic) effects on the host while the host tries to do the same to the pathogen (e.g. attack it through its immune system). The balance between these rewards and repercussions will determine the success of the pathogen, and the indirect consequences for the host for its interactions with its resources and predators. See figure 1 for the interpretation of the different types of arrow.

A possible objection against the size-based food web template that we laid out in figure 3 is that it does not accommodate pathogens and parasites. Very large herbivores and predators can have very small enemies as well. Also, our main list of six interaction types does not include host–pathogens interactions. Yet various recent papers have stated that parasites are a key factor in understanding food webs (Lafferty & Kuris 2002; Lafferty *et al*. 2006, 2008). So does our proposed framework break down at this point? We argue that it does not, as parasites can be perfectly accommodated as part of interaction webs through a combination of consumer–resource interactions, effects of species on abiotic (non-resource) conditions, responses of species to these conditions and direct non-trophic interactions among species (figure 8), i.e. through just a combination of the interaction types listed in figure 1. The key point is that a state of abiotic conditions has to be assumed, which exists *within* organisms, as a result of their physiological homeostasis (figure 8). By living inside other organisms, pathogens become decoupled from potential limitation by unfavourable external conditions to which they would be poorly adapted (being very small and ectothermic). The stoichiometric framework shown in figure 2 also still holds, because they compete with their hosts for resources, but only after their much bigger host has digested these resouces. However, figure 8 shows that parasites and pathogens cannot simply be plugged in into food webs as additional consumers—a separate network of interactions, which involves reciprocal negative direct effects between host and pathogens (of which the strength can be different for different host species), is at least required. Various implicit and explicit approaches may then be used to study their role in ecological networks.

### (d) Sampling theory for interaction webs

Interaction webs are often highly variable in space and time. Not all interactions may be present everywhere all the time (Berg & Bengtsson 2007; McCann & Rooney 2009). Rare interactions have a higher risk of not being observed than common, frequent interactions. However, rare and weak interactions may be very important in determining system dynamics (Power *et al*. 1996). This calls for the development of new, likelihood-based interaction web theory that takes the sampling nature of the data explicitly into account, and that can discriminate between alternative models and explanations (Alonso *et al*. 2006; Allesina *et al*. 2008). This approach has been successful in comparing alternative models (including different types of main process) for the determinants of community structure within trophic levels (Etienne & Olff 2005; Etienne *et al*. 2007). It can also potentially be applied to compare the relative importance of different types of interaction in parallel networks.

### (e) Conservation considerations

An important conclusion of this paper is that consumer–resource interactions are only a subset of the relevant interactions that structure most ecosystems. This implies that *managing ecosystems on the basis of consumer*–*resource interactions alone is unlikely to be sustainable*. A good case is made by the management of fishes and shellfish stocks (e.g. Piersma *et al*. 2001). This field has a long history of the development of harvesting models just based on consumer–resource interactions, (size-structured) population dynamics and food webs (Hilborn *et al*. 1995; van Kooten & Bulte 2000). Many fisheries (but not all) have been regulated on the basis of the predictions of these models, by setting harvesting quota. Yet such scientifically informed management has not prevented a general collapse of fish stocks worldwide, especially for species on higher trophic levels (Myers & Worm 2003; Worm & Myers 2004; Worm *et al*. 2005, 2006; Berkes *et al*. 2006; Heithaus *et al*. 2008). So something has gone wrong here. We suggest that a main cause of this prime failure of research–management interaction is that five out of the six main interaction types that operate in ecosystems (figure 1) have generally been ignored. Focusing on consumer–resource interactions alone may lead to surprising collapses of populations and regime shifts in ecosystem states if other, non-trophic interactions (such as dispersal, species–sediment interactions, physical interactions involving temperature) start to kick in (Weijerman *et al*. 2005; van Nes *et al*. 2007). Therefore, we think that the analysis of the relative importance and interplay among parallel interaction webs within appropriate templates (as in figure 3) is urgently needed to prevent further loss of biodiversity and impairment of ecosystem functioning worldwide.
